# Supplementary material for: Nasopharyngeal colonization by Streptococcus pneumoniae in children and adults before the introduction of the 10-valent conjugate vaccine, Paraguay
Source: PLoS One. 2023 Feb 16;18(2):e0280722. doi: 10.1371/journal.pone.0280722 (PMC9934358; doi:10.1371/journal.pone.0280722)
Supplement: Attachment — Submitted filename: Response to Reviewers.docx [file pone.0280722.s002.docx]

**Letter to Academic Editor and Revieweres**

Respectfully, all the changes made to the manuscript and the pertinent clarifications are detailed below.

**Journal Requirements:**

1. Please ensure that your manuscript meets PLOS ONE's style requirements, including those for file naming. The PLOS ONE style templates can be found at

https://journals.plos.org/plosone/s/file?id=wjVg/PLOSOne_formatting_sample_main_body.pdf and

https://journals.plos.org/plosone/s/file?id=ba62/PLOSOne_formatting_sample_title_authors_affiliations.pdf

Reponse:

We have reviewed the PLOS ONE style requirements.

2. Please ensure that you include a title page within your main document. We do appreciate that you have a title page document uploaded as a separate file, however, as per our author guidelines (http://journals.plos.org/plosone/s/submission-guidelines#loc-title-page) we do require this to be part of the manuscript file itself and not uploaded separately.

Could you therefore please include the title page into the beginning of your manuscript file itself, listing all authors and affiliations.

Reponse:

We have included the title page at the beginning of the manuscript file, listing all authors and affiliations.

3. We note that the grant information you provided in the ‘Funding Information’ and ‘Financial Disclosure’ sections do not match.

When you resubmit, please ensure that you provide the correct grant numbers for the awards you received for your study in the ‘Funding Information’ section.

Reponse:

This research has not received any specific scholarship from public sector agencies of the Ministry of Public Health and Social Welfare, PNEI-PAI or the LCSP. Therefore they do not have a grant number and we have decided to withdraw this information.

4. In your Data Availability statement, you have not specified where the minimal data set underlying the results described in your manuscript can be found. PLOS defines a study's minimal data set as the underlying data used to reach the conclusions drawn in the manuscript and any additional data required to replicate the reported study findings in their entirety. All PLOS journals require that the minimal data set be made fully available. For more information about our data policy, please see http://journals.plos.org/plosone/s/data-availability.

Upon re-submitting your revised manuscript, please upload your study’s minimal underlying data set as either Supporting Information files or to a stable, public repository and include the relevant URLs, DOIs, or accession numbers within your revised cover letter. For a list of acceptable repositories, please see http://journals.plos.org/plosone/s/data-availability#loc-recommended-repositories. Any potentially identifying patient information must be fully anonymized.

Important: If there are ethical or legal restrictions to sharing your data publicly, please explain these restrictions in detail. Please see our guidelines for more information on what we consider unacceptable restrictions to publicly sharing data: http://journals.plos.org/plosone/s/data-availability#loc-unacceptable-data-access-restrictions. Note that it is not acceptable for the authors to be the sole named individuals responsible for ensuring data access.

We will update your Data Availability statement to reflect the information you provide in your cover letter.

Reponse:

We have uploaded the minimum set of data underlying your study as supporting data files.

Also, the minimal dataset underlying the results described in the manuscript can be found at: 10.6084/m9.figshare.21089815 (The DOI becomes active when the item is published).

**Additional Editor Comments:**

The study period is not clear from the text as the authors have mentioned it to be between June and December 2012 in abstract while in materials and methods it has been mentioned to be from April through December 2012.

Reponse: The samples were collected from april to july 2012.

The authors need to clarify why the exclusion criteria includes any documented dose of pneumococcal vaccine given the fact that PCV-10 was introduced in December 2012 and the study period is before the introduction of PCV-10.

Reponse: The pneumococcal vaccine data was collected because the PCV7 was probably already licensed in Paraguay and available in the private sector.

The authors need to clarify the use of CLSI 2020 guidelines for the study done in the year 2012.

Reponse: The pneumococcal isolation was done and the isolates stored. However, the MIC testing was performed only in 2020 at CDC.

Responses to Reviewer #1

1. The authors of the present manuscript intends to find S. pneumoniae baseline prevalence, serotype distribution, and antibiotic resistance patterns in healthy children aged 2 to 59 months and adults ≥60 years of age prior to the introduction of PCV10 in the national childhood immunization program in Paraguay. Although the study seems interesting at the first glance, there some key issues related to the selection of subjects and statistical analyses which need to be addressed by the authors. Moreover, the study was conducted almost a decade ago. It is not clear whether the study is part of an ongoing surveillance program or a standalone project. Without the follow up data after introduction of the PCV-10, the findings of the study may not be impactful.

Reponse:

Although it was planned to carry out the post-vaccination study, unfortunately, for financial reasons we have not been able to carry it out. In the same way, we believed it convenient to present the results of the pre-vaccination study, since this is the only study that has been carried out in healthy carriers in Paraguay.

Reponse: The study is part of an ongoing surveillance program. Although it was planned to carry out the post-vaccination study, unfortunately, for financial reasons we have not been able to carry it out. In the same way, we believed it convenient to present the results of the pre-vaccination study, since this is the only study that has been carried out in healthy carriers in Paraguay.

2. Abstract

Lines 17-19: Colonized children were more likely to share a bedroom (P=0.03) and have a history of respiratory infection (P=0.095) or pneumococcal infection (P=0.06) compared to non-colonized children,….

Comment: The above statistics are not worth mentioning because for variables like history of respiratory infection and pneumococcal infection, the p values are higher that is assumed significant for the present study (i.e. <0.05). Moreover, sharing of bedroom has not been found significant (as mentioned under Results) after adjusting for congenital disease, pneumonia, and smokers present in the household.

Reponse:

We have eliminated the statistics being as follows:

Colonized children were more likely to share a bedroom, have a history of respiratory infection or pneumococcal infection compared to non-colonized children. No associations were found in adults. However, no significant associations were found in children and neither in adults.

3. Introduction

Lines 30-32: According to the World Health Organization (WHO), of the estimated 5.83 million deaths among children <5 years of age globally in 2015, 294,000 were caused by pneumococcal infections.

Comment: Please add the uncertainty interval within parenthesis after the estimated figure of 294,000.

Reponse:

We have added the range of uncertainty: (uncertainty range [UR], 192 000–366 000)

4. Lines 32-34: The burden of pneumococcal disease is high in children, older adults, and those who are immunosuppressed, and it occurs in high-, low-, and middle-income countries.

Comment: The authors may consider slightly modifying the later part of the above sentence.

…..and most of the deaths occur in developing countries’ instead of ‘….and it occurs in high-, low-, and middle-income countries’. Reference- https://www.cdc.gov/pneumococcal/global.html

Reponse: we have modified according to suggestion “and most of the deaths occur in developing countries”

5. Lines 39-42: Children are more commonly colonized with S. pneumoniae than adults, and the highest colonization rates are found among children less than five years of age, which corresponds precisely with the highest incidence of pneumococcal disease.

Comment: The statistical estimate for carriage related prevalence among both children and adults from reference no. 07 by Brooks et al. could be incorporated for the comparative depiction between the mentioned groups.

Reponse:

We have added the requested prevalence, in children 20-50% and adults 5-20%.

6. Lines 45-46: Children have been suggested to be the source of transmission in the household.

Comment: Although true, the above statement seems incomplete without mentioning the other findings from the cited literature. Like, children are also found to be involved more in community transmission than adults.

Reponse:

We have added according to the mentioned literatura and suggested “also be involved more in community transmission than adults”.

7. Materials and methods

Lines 63 to 76: We enrolled all children aged 2 to 59 months and adults ≥60 years of age………………………… through the Ministry of Health and Social Welfare in the health units and immunization centers in Paraguay.

Comment: The study design is poorly understood. The authors should make a flow diagram for allowing better understanding of the selection of subjects.

There are several queries which need to be clarified.

a) As per the title and aim of the present manuscript the authors intend to demonstrate the pneumococcal colonization prevalence, serotype, distribution, etc. before the introduction of PCV-10 vaccine. The study subjects were included from April to December 2012. As per the authors, the PCV 10 was introduced in December 2012.

However, as per a previously published study, PCV10 has been introduced much earlier than the study period.

Kieninger MP, Caballero EG, Sosa AA, Amarilla CT, Jáuregui B, Janusz CB, Clark AD, Castellanos RM. Cost-effectiveness analysis of pneumococcal conjugate vaccine introduction in Paraguay. Vaccine. 2015 May 7;33 Suppl 1:A143-53.

Reponse:

According to Castellanos RM. Cost-effectiveness analysis of pneumococcal conjugate vaccine introduction in Paraguay. Vaccine. 2015 May 7;33 Suppl 1:A143-53.

TRIVAC model version 2.0 is a tool that evaluates vaccine impact and incremental costeffectiveness ratios (ICER), providing a coherent and transparent framework for each vaccine, with comparable and standardized outcomes. Utilizing parameters, such as demography, disease burden, vaccine costs, vaccine coverage, vaccine efficacy, vaccine serotype coverage, health service utilization and costs.

In order to estimate coverage per dose in the year of introduction, EPI information on Pentavalent and MMR1 dose coverage over the last 5 years was used.

Therefore, for this mentioned study, the real application of any PCV has not been implemented.

b)The relevance of the following sentence is not at all understood. “PCV10 was introduced for all eligible children (two primary doses in children <1 year and one booster at one year of age, in December 2012”

The PCV-10 vaccination schedule followed in Paraguay can be incorporated as supplementary file. If the vaccine has been introduced in December 2012 for the first time, how can the booster be considered for few children at the same time.

Reponse:

We have modified according to suggestion:

“In December 2012, the Ministry of Health and Social Welfare of Paraguay introduced PCV10 to the regular vaccination schedule of the Expanded Program on Immunizations, using the three-dose schedule (Scheme 2 + 1): two primary doses in children <1 year ( 2 to 11 months) and a booster at one year of age (12 months or 6 months after the second dose)”.

8. Lines 81-82: Documentation of any dose of pneumococcal vaccine or incomplete information to accurately determine immunization status.

Comment: Some of the important exclusion criteria have not been considered. For example, recent administration of antibiotics which might influence the S. pneumoniae colonizations. Please justify.

Reponse:

We agree with the reviewer that recent exposure to antibiotics may affect the colonization results either leading to lower detection of colonization or detection of resistance pneumococci. Many colonization studies do not exclude patients on antibiotics since you can still detect pneumococci among patients with recent antibiotic exposure.

9. Line 97: Pneumococcal isolates were serotyped by Quellung reaction and multiplex PCR.

Comment: Modify the above sentence like “The serotypes of the pneumococcal isolates were identified by Quellung reaction and multiplex PCR”. The methodology of multiplex PCR should be mentioned or referenced.

Reponse:

We have modified according to suggestion: The serotypes of the pneumococcal isolates were identified by Quellung reaction and multiplex PCR.

And we added the referencePai R, Gertz RE, Beall B. Sequential multiplex PCR approach for determining capsular serotypes of Streptococcus pneumoniae isolates. J Clin Microbiol. 2006; 44(1):124-31. doi: 10.1128/JCM.44.1.124-131.2006.

10. Lines 106-114: Pneumococcal isolates were tested for susceptibility…… breakpoints of the 2020 Clinical and Laboratory Standards Institute (CLSI) guidelines.

Comment: The reviewer feels curious to know as when the susceptibility testing was actually performed. Because the samples were collected in 2012 and the interpretation of the test was done as per the CLSI guidelines published in 2020. Moreover, was susceptibility testing individually for all morphotypes of the colonies were performed if more than one colony type was isolated (as it is done for serotyping)?

Reponse:

The pneumococcal isolation was done and the isolates stored. However, the MIC testing was performed only in 2020 at CDC.

Yes, all morphotypes of colonies with diferente serotypes were tested for antimicrobial susceptibility test by broth microdilution.

11. Lines 170-173: Children who shared a bedroom had 2.3 times increased odds of being a pneumococcal carrier compared to those who did not share a bedroom (35% vs.18%, p=0.03). However, this association was no longer significant after adjusting for congenital disease, pneumonia, and smokers present in the household.

Comment: The adjusted p values could not be found in the Table 02.

How was the adjustment carried out? Was a multivariate analysis performed? More details on the statistical analyses are absent under Materials and Methods.

Reponse:

We have added to methods: “Multivariable analysis, where appropriate, was performed to evaluate independent risk factors associated with pneumococcal colonization. Variables with P values< 0.30 in univariable analysis were included as candidate variables for the model. Multivariable analyses was not conducted if univariable analyses showed <2 variables with P<0.30 for the outcome of interest”.

We have modified the results:

However, this association was no longer significant after adjusting for congenital disease (p=0.569), pneumonia (p=0.118), and smokers present in the household (p=0.194). In adults, no statistically significant associations with pneumococcal carriage were observed.

Table 3. Multivariable analysis for pneumococcal colonization risk factors among children (n=245).

| **Risk factor** | **OR (95% CI)** | ***P-adjusted*** |
| --- | --- | --- |
| Share room | 2.38 (1.03-5.49) | *0.072* |
| Smokers present | 1.31 (0.93-1.84) | *0.194* |
| Congenital condition | 1.54 (0.69-3.45) | *0.569* |
| Illness/Pneumonia | 2.18 (0.95-5.02) | *0.118* |

12. Lines 187-195: In children, multidrug resistance (MDR) was found in 19/245 (7.7%) of……….. and 2/24 (8.3%) of erythromycin-clindamycin-trimethoprim/sulfamethoxazole-tetracycline.

Comment: Please consider rephrasing of the two sentences mentioned in the above paragraph. It will be better if the authors present the susceptibility profile in form of graphs (showing susceptibility/resistance proportions) separately in children and adults. The above description of MDR (presumably as at least one member of ≥ 3 classes of antibiotics) seems to be complex. The authors should find easier way of depiction of the above statistics either in tables or figures.

Reponse: We have considered reformulating the two mentioned sentences, remaining as follows:

In children, of the 245 pneumococcal isolates, 19 (7.7%) were resistant to erythromycin-trimethoprim/sulfamethoxazole-tetracycline, 18 (7.3%) were resistant to erythromycin-trimethoprim/sulfamethoxazole-tetracycline-cefuroxime, 18 (7.3%) were resistant to erythromycin-clindamycin-trimethoprim/sulfamethoxazole-tetracycline, and 6 (2.4%) were resistant to erythromycin-trimethoprim/sulfamethoxazole-tetracycline-chloranphenicol. In adults, of the 24 pneumococcal isolates, 3 (12.5%) were resistant to erythromycin-trimethoprim/sulfamethoxazole-tetracycline, 2 (8.3%) were resistant to erythromycin-trimethoprim/sulfamethoxazole-tetracycline-cefuroxime, and 2 (8.3%) were resistant to erythromycin-clindamycin-trimethoprim/sulfamethoxazole-tetracycline.

Table 4. Distribution of antimicrobial resistance by serotypes.

| **2 - 59 months** | | | | | | | | | | | | | | | | |
| --- | --- | --- | --- | --- | --- | --- | --- | --- | --- | --- | --- | --- | --- | --- | --- | --- |
| **Antibiotics** | **PCV10** | | | | | | | | | | **PCV13** | | | | ***p*** | |
|  | **4** | **6B** | **9V** | **14** | **18C** | **19F** | **23F** | **1** | **5** | **7F** | **3** | **6A** | **19A** |  | |  |
| Penicillin G | 0 | 0 | 0 | 0 | 0 | 0 | 0 | 0 | 0 | 0 | 0 | 0 | 0 | *0.923* | |  |
| Ceftriaxone | 0 | 0 | 0 | 0 | 0 | 0 | 0 | 0 | 0 | 0 | 0 | 0 | 0 | *0.504* | |  |
| Azithromycin | 0 | 7(16.67) | 0 | 7(41.18) | 0 | 18(56.25) | 4(20.00) | 0 | 0 | 0 | 0 | 7(16.67) | 0 | *0.002* | |  |
| Tetracycline | 0 | 6(14.29) | 0 | 7(41.18) | 1(25.00) | 16(50.00) | 5(25.00) | 0 | 0 | 0 | 1(50.00) | 0 | 0 | *<0.001** | |  |
| Erythromycin | 0 | 7(16.67) | 0 | 7(41.18) | 0 | 18(56.25) | 4(20.00) | 0 | 0 | 0 | 0 | 7(4375) | 0 | *0.002* | |  |
| TMP–SMX | 0 | 8(19.05) | 3(75.00) | 11(64.71) | 0 | 18(56.25) | 7(35.00) | 0 | 0 | 0 | 0 | 1(6.25) | 0 | *0.001* | |  |
| Clindamycin | 0 | 6(14.29) | 1(25.00) | 1(5.88) | 1(25.00) | 16(50.00) | 0 | 0 | 0 | 0 | 0 | 0 | 0 | *0.221* | |  |
| Chloramphenicol | 0 | 2(4.76) | 0 | 0 | 0 | 1(3.13) | 2(10.00) | 0 | 0 | 0 | 0 | 0 | 0 | *0.030* | |  |
| AMC | 0 | 0 | 0 | 0 | 0 | 1(3.13) | 0 | 0 | 0 | 0 | 0 | 0 | 0 | *1.000* | |  |
| Cefuroxime | 0 | 2(4.76) | 0 | 4(23.53) | 0 | 16(50.00) | 3(15.00) | 0 | 0 | 0 | 0 | 0 | 0 | *0.523* | |  |
| **≥ 60 years** | | | | | | | | | | | | | | | | |
| Penicillin G | 0 | 0 | 0 | 0 | 0 | 0 | 0 | 0 | 0 | 0 | 0 | 0 | 0 | *-* | |  |
| Ceftriaxone | 0 | 0 | 0 | 0 | 0 | 0 | 0 | 0 | 0 | 0 | 0 | 0 | 0 | *-* | |  |
| Azithromycin | 0 | 0 | 0 | 0 | 0 | 2(100) | 0 | 0 | 0 | 0 | 0 | 2(66.67) | 0 | *0.144* | |  |
| Tetracycline | 0 | 0 | 0 | 1(100) | 0 | 2(100) | 0 | 0 | 0 | 0 | 0 | 1(33.33) | 0 | *0.107* | |  |
| Erythromycin | 0 | 0 | 0 | 0 | 0 | 2(100) | 0 | 0 | 0 | 0 | 0 | 2(66.67) | 0 | *0.144* | |  |
| TMP–SMX | 0 | 0 | 0 | 0 | 0 | 2(100) | 0 | 0 | 0 | 0 | 0 | 1(33.33) | 0 | *0.648* | |  |
| Clindamycin | 0 | 0 | 0 | 0 | 0 | 0 | 0 | 0 | 0 | 0 | 0 | 1(100) | 0 | *0.823* | |  |
| Chloramphenicol | 0 | 0 | 0 | 0 | 0 | 0 | 0 | 0 | 0 | 0 | 0 | 0 | 0 | *0.904* | |  |
| AMC | 0 | 0 | 0 | 0 | 0 | 0 | 0 | 0 | 0 | 0 | 0 | 0 | 0 | *-* | |  |
| Cefuroxime | 0 | 2(66.67) | 0 | 0 | 0 | 2(100) | 0 | 0 | 0 | 0 | 0 | 0 | 0 | *0.144* | |  |

TMP–SMX: Trimethoprim-sulfamethoxazole

AMC: Amoxicillin/Clavulanic Acid

13. The manuscript should be checked and edited by a native English speaker.

The collected data is almost a decade old. How will the authors justify the importance of the findings in recent time (given the colonization pattern have been likely changed over the past decade)? The reviewer is of the opinion that the authors may conduct a follow up surveillance to depict the changes in the colonization after 10 years of PCV-10 introduction in Paraguay.

Reponse:

The authors agree on the importance of conducting follow-up surveillance to represent changes in colonization 10 years after the introduction of PCV-10 in Paraguay.

**General comments:**

The manuscript should be checked and edited by a native English speaker.

The collected data is almost a decade old. How will the authors justify the importance of the findings in recent time (given the colonization pattern have been likely changed over the past decade)? The reviewer is of the opinion that the authors may conduct a follow up surveillance to depict the changes in the colonization after 10 years of PCV-10 introduction in Paraguay.

Reponse:

We agree with the reviewer. Although it was planned to carry out the post-vaccination study, unfortunately for economic reasons we have not been able to carry it out. Similarly, we thought it appropriate to present the results of the pre-vaccination study, since this is the only study that has been carried out in healthy carriers in Paraguay. We will do our best to carry out a postvaccinal colonization study.

**Responses to Reviewer #2**

**Reviewer #2**: The manuscript is written in crisp language and focused to answer the research question. But this study should be followed by another follow up study to assess the response of nationwide PCV 10 vaccination on nasopharyngeal colonization of streptococcus pneumoniae.

Reponse:

We agree with the reviewer. Although it was planned to carry out the post-vaccination study, unfortunately for economic reasons we have not been able to carry it out. Similarly, we thought it appropriate to present the results of the pre-vaccination study, since this is the only study that has been carried out in healthy carriers in Paraguay.

King regards

María Eugenia León
