# Supplementary material for: Nasopharyngeal colonization by Streptococcus pneumoniae in children and adults before the introduction of the 10-valent conjugate vaccine, Paraguay
Source: PLoS One. 2023 Feb 16;18(2):e0280722. doi: 10.1371/journal.pone.0280722 (PMC9934358; doi:10.1371/journal.pone.0280722)
Supplement: Attachment — Submitted filename: Response to Reviewers.docx [file pone.0280722.s003.docx]

**Reponse to reviewers:**

1. Journal Requirements:

Please review your reference list to ensure that it is complete and correct. If you have cited papers that have been retracted, please include the rationale for doing so in the manuscript text, or remove these references and replace them with relevant current references. Any changes to the reference list should be mentioned in the rebuttal letter that accompanies your revised manuscript. If you need to cite a retracted article, indicate the article’s retracted status in the References list and also include a citation and full reference for the retraction notice.

Reponse: We have reviewed the reference list and it is complete. No new articles have been cited.

2. Multivariable analysis, where appropriate, was performed to evaluate independent risk factors associated with pneumococcal colonization.

Comment: Please specify the method that was adopted for multivariable analysis.

Reponse: The method adopted for the multivariate analysis is specified as follows:

“Multivariable analysis, was performed to evaluate independent risk factors associated with pneumococcal colonization. Separate multivariable models were constructed for each outcome using automated stepwise logistic regression (congenital disease, pneumonia and presence of smokers in the household)”.


3. The serotypes found most frequently with resistance to trimethoprim/sulfamethoxazole, tetracycline, erythromycin, cefuroxime, and clindamycin were 19F, 14, and 6B (Table 4).

Comment: The inference drawn in the sentence just before Table 4 does not completely match with the statistics mentioned in the table. Hence, the above sentence should be deleted.

Reponse: Because the inference drawn in the sentence just before Table 4 does not fully match the statistics mentioned in the table.

Deleted sentence: The serotypes found most frequently with resistance to trimethoprim/sulfamethoxazole, tetracycline, erythromycin, cefuroxime, and clindamycin were 19F, 14, and 6B.

4. Reviewer #1: The authors have responded to the queries or comments satisfactorily. However the following needs to be incorporated in the manuscript: under material and methods the study period has still not been mentioned clearly. Kindly give specific study period details.

Reponse: In order to provide specific details of the study period, the following is added in material and methods:

Setting, design and study period

As part of the national surveillance program for meningitis and pneumonia, a cross-sectional study was carried out, whose study period was from 2012 to 2020.

5. PLOS authors have the option to publish the peer review history of their article (what does this mean?). If published, this will include your full peer review and any attached files.

If you choose “no”, your identity will remain anonymous but your review may still be made public.

Reponse: Yes, I want your identity to be public for this peer review
